# Genome-wide trait-trait dynamics correlation study dissects the gene regulation pattern in maize kernels

**DOI:** 10.1186/s12870-017-1119-y

**Published:** 2017-10-16

**Authors:** Xiuqin Xu, Min Wang, Lianbo Li, Ronghui Che, Peng Li, Laming Pei, Hui Li

**Affiliations:** 1 School of Biological and Science Technology, University of Jinan, Jinan, 250022 China; 2 National Maize Improvement Center of China, Key Laboratory of Crop Genomics and Genetic Improvement, China Agricultural University, Beijing, 100193 China

**Keywords:** Maize, Liquid association, Trait-trait correlation dynamics, Carotene, Oil

## Abstract

**Background:**

Dissecting the genetic basis and regulatory mechanisms for the biosynthesis and accumulation of nutrients in maize could lead to the improved nutritional quality of this crop. Gene expression is regulated at the genomic, transcriptional, and post-transcriptional levels, all of which can produce diversity among traits. However, the expression of most genes connected with a particular trait usually does not have a direct association with the variation of that trait. In addition, expression profiles of genes involved in a single pathway may vary as the intrinsic cellular state changes. To work around these issues, we utilized a statistical method, liquid association (LA) to investigate the complex pattern of gene regulation in maize kernels.

**Results:**

We applied LA to the expression profiles of 28,769 genes to dissect dynamic trait-trait correlation patterns in maize kernels. Among the 1000 LA pairs (LAPs) with the largest LA scores, 686 LAPs were identified conditional correlation. We also identified 830 and 215 LA-scouting leaders based on the positive and negative LA scores, which were significantly enriched for some biological processes and molecular functions. Our analysis of the dynamic co-expression patterns in the carotene biosynthetic pathway clearly indicated the important role of *lcyE, CYP97A, ZEP1*, and *VDE* in this pathway, which may change the direction of carotene biosynthesis by controlling the influx and efflux of the substrate. The dynamic trait-trait correlation patterns between gene expression and oil concentration in the fatty acid metabolic pathway and its complex regulatory network were also assessed. 23 of 26 oil-associated genes were correlated with oil concentration conditioning on 580 LA-scoutinggenes, and 5% of these LA-scouting genes were annotated as enzymes in the oil metabolic pathway.

**Conclusions:**

By focusing on the carotenoid and oil biosynthetic pathways in maize, we showed that a genome-wide LA analysis provides a novel and effective way to detect transcriptional regulatory relationships. This method will help us understand the biological role of maize kernel genes and will benefit maize breeding programs.

**Electronic supplementary material:**

The online version of this article (10.1186/s12870-017-1119-y) contains supplementary material, which is available to authorized users.

## Background

Maize is one of the most widely grown crops in the world and also is a very important model organism [1]. Carotenes and fatty acids are two important nutrients in maize kernels. Understanding the genetic architecture and regulation mechanism of their biosynthesis and accumulation will be of great value for improving the nutritional quality of maize. Based on linkage analysis, map-based cloning, and candidate gene association mapping methods, there are more than 100 loci or candidate genes involved in maize kernel oil and carotene accumulation [2–6]. With the completion of a high-quality maize genome sequence and the availability of high-throughput phenotyping technologies, genome-wide association (GWA) studies have quickly become a powerful, general tool for identifying alleles and loci responsible for natural variation in maize [7]. Recently, an association mapping panel of 500 maize inbred lines and 560,000 polymorphisms with minor allele frequency (MAF) ≥ 0.05 was used to identify 74 loci significantly associated with kernel oil concentration and fatty acid composition [8]. Moreover, the transcription profiles of maize kernel development have been generated for two maize inbred lines, resulting in the identification of differentially expressed genes and the functional characterization of genes involved in kernel developmental pathways [9, 10]. Fu et al. characterized a large-scale gene regulatory network in maize kernels using RNA-sequencing (RNA-seq) in 368 inbred lines, which identified expression quantitative trait loci (e-QTLs) as well as the relationship between these e-QTLs and their targets [11]. These studies have led to a deeper understanding of carotene and oil biosynthesis pathways, including the genes involved and their regulation.

Phenotypic variation is regulated at the genomic, transcriptional, and post-transcriptional levels [12]. As an example of genomic-level regulation, a phenylalanine insertion in maize acyl-CoA diacylglycerol acyl transferase (DGAT), which catalyzes the final step in the Kennedy pathway for triacylglycerol (TAG) biosynthesis, alters enzyme activities and is responsible for increased oil and oleic-acid contents [13]. *crtRB1*, which encodes β-carotene hydroxylase, is an example of the importance of transcript abundance during the control of carotenoid profiles. *crtRB1* alleles associated with reduced transcript expression correlate with high β-carotene concentrations [4]. Transgenic results confirmed that *LEAFY COTYLEDON1* (*ZmLEC1*) and *WRINKLED1* (*ZmWR11*) in maize, both of which encode transcription factors, regulate oil concentration variation at the transcriptional level [14]. Thus, differences in gene expression may account for a substantial proportion of the variation in traits, especially for quantitative traits. However, the expression level of trait-associated genes does not correlate with the phenotypic variation of target traits at *P* value <0.01 [8]. In addition, highly co-expressed genes may be involved in the same biological process or metabolic pathway [15, 16], but the expression profiles of most genes in the same pathway are often uncorrelated [17]. Recent studies have demonstrated that the co-regulation pattern of two genes is affected by the expression levels of third genes or genetic variations in yeast and humans [18–23].

Liquid association (LA) theory offers a scoring system to guide a genome-wide search for the critical cellular players that may affect the co-expression pattern of any gene pair (*X*, *Y*) [20, 21]. Thus, LA is an extension of the traditional correlation measure, which is effectively used in gene expression studies for identifying the mediator genes in pathways or metabolic pathways in yeast [21, 22]. LA is also used to find candidate genes that intervene, confound, and weaken the drug-gene correlation [18, 19]. In general, the LA method is a recently developed tool for understanding the biological roles of genes that had not previously been applied in plants.

In this study, two datasets were used. One is a gene expression dataset of poly(A) transcripts collected from kernels at 15 days after pollination from all 368 lines sequenced using 90-bp paired-end Illumina sequencing with libraries of 200-bp inserts [11]. The other dataset contains the oil concentration collected from the kernels of the 368 maize inbred lines [8]. Based on these data, we carried out a full genome-wide study on more than 1.10 × 10^12^ gene triplets to investigate trait-trait dynamic correlations conditioning on LA-scouting genes. Our objectives were to (1) capture the dynamic co-expression pattern between genes while controlling for the constantly varying expression of genes, (2) focus on a new attempt to study the trait-trait correlation patterns between gene pairs in the metabolic pathway or between a gene and a quantitative trait by LA, (3) use the LA method to study the direction of gene regulation in pathways and (4) find more candidate genes in a biosynthesis pathway or metabolic pathway.

## Results

### Genome-wide results of the co-expression dynamic pattern of gene pairs

**Fig. 1:**
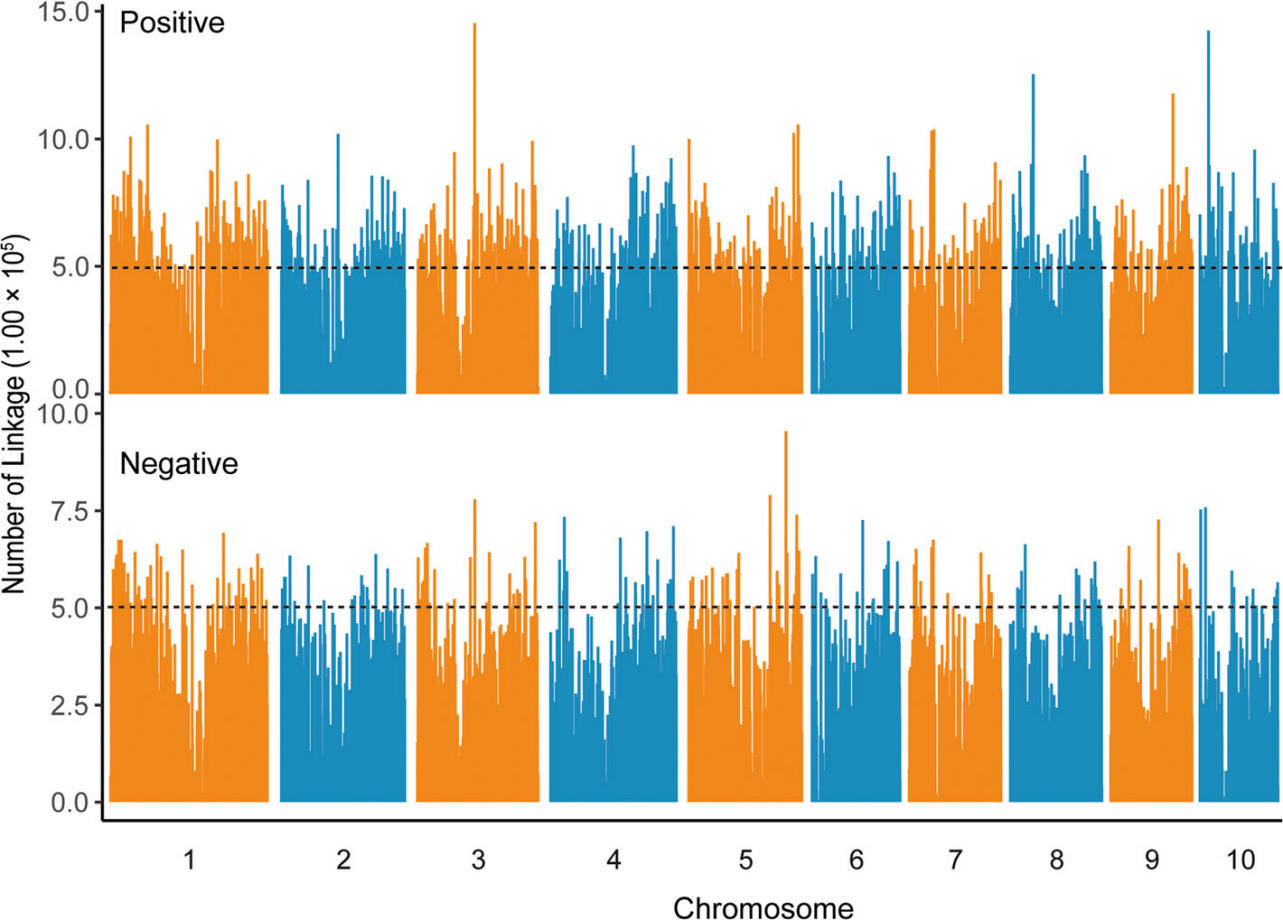
The genome-wide results of LA in maize. The LA-scouting gene frequency for positive and negative LA scores. The cutoff for *P* value of LA is 1.00 × 10^−5^. Broken dashed lines indicate the LA-scouting gene frequency (5.00 × 10^5^)

From 28,769 annotated genes in 368 maize lines, we selected 24,907 genes with a missing rate of <20% for this study [11]. We conducted a genome-wide search for the cellular players that may affect the co-expression pattern of any two gene pairs using LA theory. Then we computed two genome-wide distributions of LA linkage scores, one for positive scores and the other for negative scores, based on a total of 1.10 × 10^12^ triplets. As the LA linkage analysis consisted of a large number of gene pairs, the majority of which are probably biologically unrelated, the LA linkage scores were highly susceptible to random chance. We have performed a series of permutations from one million to one hundred million. And we found that the LA thresholds are similar regardless of the number of permutation times at 1.00 × 10^−5^ quantile (Additional file 1: Table S1). Using one million times permutation, we obtained one reference distribution for the positive LA linkage scores and the other for the negative LA linkage scores (see Methods). According to the quantile-to-quantile plot of genome-wide LA scores versus randomly generated LA scores, we found a global linear pattern with an upward shift for the genome-wide positive LA linkage scores and a downward shift for the negative scores (Additional file 2: Figure S1). Using the 1.00 × 10^−5^ quantile from the reference distribution as the cutoff, we obtained 1.60 × 10^9^ and 1.10 × 10^9^ gene triplets with positive and negative LA scores, respectively (Fig. 1 and Additional file 3: Table S2). And the modified liquid association (MLA) method was used to define the LA-scouting gene in each triplet. The false discovery rate (FDR) for positive and negative LA findings were 0.72% and 0.98%, respectively (Additional file 4: Figure S2 and Additional file 5: Table S3).

We checked the LAPs (which consisted of a gene pair, *X* and *Y*) with exceptionally positive or negative LA scores and focused on a small portion of the 1000 high-scoring LAPs. Among these 1000 LAPs, 686 LAPs were correlated only conditioning on the gene expression levels of LA-scouting genes (*r* < 0.17 and LA > 0.60). The 50 LAPs with the highest LA scores from the original group of 686 are shown in Table 1 (At the end of the paper). We then annotated the top 50 triplets with the highest LA scores (Additional file 6: Table S4) and found that some of them are functionally associated with or involved in the same metabolic pathways, which were highlighted in Table S3**.** For example, the triplet *GRMZM2G033626*, *GRMZM2G388576*, and *GRMZM2G446426* encode Mov34/MPN/PAD-1 family proteins, which function as the ubiquitin isopeptidase/deubiquitinase in the ubiquitin-based signaling and protein turnover pathways in eukaryotes [24]. Another gene pair, *GRMZM2G064133* and *GRMZM2G056393*, which encode translation initiation factor 3G1 and translation elongation factor EFG/EF2 protein, respectively, which are both involved in protein translation process.

**Table 1:** The top 50 triplets with the highest LA scores in a genome-wide LA analysis

*X*	*Y*	*Z*	LA	(*Y*,*Z*)|*X*	(*X*,*Z*)|*Y*	(*X*,*Y*)|*Z*
*AF546188.1_FG003*	*AF546188.1_FG002*	*AF546188.1_FG001*	−0.72	−0.03	−0.42	−0.43
*GRMZM2G172980*	*GRMZM2G134020*	*GRMZM2G122520*	−0.71	−0.25	−0.34	−0.47
*GRMZM2G172980*	*GRMZM2G134020*	*GRMZM2G161750*	−0.70	−0.19	−0.30	−0.40
*GRMZM2G172980*	*GRMZM2G134020*	*GRMZM2G150524*	−0.69	−0.23	−0.29	−0.43
*GRMZM2G172980*	*GRMZM2G134020*	*GRMZM2G156578*	−0.69	−0.23	−0.28	−0.38
*GRMZM2G172980*	*GRMZM2G134020*	*GRMZM5G813474*	−0.69	−0.24	−0.29	−0.42
*GRMZM2G172980*	*GRMZM2G134020*	*GRMZM2G093035*	−0.69	−0.28	−0.32	−0.42
*GRMZM2G172980*	*GRMZM2G134020*	*GRMZM2G483598*	−0.68	−0.27	−0.30	−0.40
*GRMZM2G172980*	*GRMZM2G134020*	*GRMZM5G837022*	−0.68	−0.28	−0.29	−0.45
*GRMZM2G172980*	*GRMZM2G134020*	*GRMZM5G822819*	−0.68	−0.20	−0.25	−0.45
*GRMZM2G172980*	*GRMZM2G134020*	*GRMZM2G003461*	−0.67	−0.24	−0.28	−0.37
*GRMZM2G033626*	*GRMZM2G446426*	*GRMZM2G388576*	0.67	0.20	0.41	0.44
*GRMZM2G009598*	*GRMZM2G056099*	*GRMZM2G040079*	0.66	0.30	0.35	0.36
*GRMZM2G181362*	*GRMZM2G046932*	*GRMZM2G008226*	−0.65	−0.25	−0.29	−0.33
*GRMZM2G064133*	*GRMZM2G082271*	*GRMZM2G008226*	0.65	0.22	0.31	0.35
*GRMZM2G096010*	*GRMZM5G891343*	*GRMZM2G396773*	0.65	0.15	0.33	0.42
*GRMZM2G008607*	*GRMZM2G146283*	*GRMZM2G090869*	−0.65	−0.30	−0.33	−0.34
*GRMZM2G009598*	*GRMZM2G156818*	*GRMZM2G101635*	0.64	0.14	0.29	0.31
*GRMZM2G025703*	*GRMZM2G180612*	*GRMZM2G146283*	0.64	0.31	0.32	0.33
*GRMZM2G010797*	*GRMZM2G035118*	*GRMZM2G060611*	0.64	0.30	0.32	0.34
*GRMZM2G009598*	*GRMZM2G056099*	*GRMZM2G321753*	0.64	0.28	0.37	0.38
*GRMZM2G126821*	*GRMZM2G082271*	*GRMZM2G008226*	0.64	0.22	0.33	0.36
*GRMZM2G009598*	*GRMZM2G101635*	*GRMZM2G321753*	0.64	0.17	0.33	0.36
*GRMZM5G821988*	*GRMZM2G179024*	*GRMZM2G072894*	−0.64	−0.19	−0.30	−0.35
*GRMZM2G119483*	*GRMZM2G035118*	*GRMZM2G046932*	0.64	0.29	0.34	0.34
*GRMZM2G172980*	*GRMZM2G134020*	*GRMZM5G884722*	−0.64	−0.25	−0.33	−0.43
*GRMZM2G064133*	*GRMZM2G056393*	*GRMZM2G008226*	0.64	0.24	0.33	0.35
*GRMZM2G010797*	*GRMZM5G876621*	*GRMZM2G060611*	0.64	0.25	0.33	0.35
*GRMZM2G010037*	*AC196475.3_FG005*	*GRMZM2G032852*	0.64	0.19	0.33	0.35
*GRMZM2G375222*	*GRMZM2G179024*	*GRMZM2G072894*	−0.64	−0.17	−0.33	−0.35
*GRMZM2G009598*	*GRMZM2G101635*	*GRMZM2G467169*	0.63	0.17	0.29	0.34
*GRMZM2G009598*	*GRMZM2G035395*	*GRMZM2G056099*	0.63	0.23	0.34	0.34
*GRMZM2G009598*	*GRMZM2G056099*	*GRMZM2G447745*	0.63	0.26	0.32	0.33
*GRMZM2G009598*	*GRMZM2G056099*	*GRMZM2G127632*	0.63	0.30	0.33	0.36
*GRMZM2G009598*	*GRMZM2G101635*	*GRMZM2G168096*	0.63	0.19	0.29	0.38
*GRMZM2G009598*	*GRMZM2G056099*	*GRMZM2G168096*	0.63	0.28	0.33	0.36
*GRMZM2G025703*	*GRMZM2G040207*	*GRMZM2G401050*	0.63	0.21	0.33	0.37
*GRMZM2G009598*	*GRMZM2G056099*	*GRMZM2G104047*	0.63	0.27	0.32	0.35
*GRMZM2G126821*	*GRMZM2G094123*	*GRMZM2G008226*	0.63	0.25	0.33	0.36
*GRMZM2G174479*	*GRMZM2G147671*	*GRMZM2G177599*	−0.63	−0.35	−0.36	−0.37
*GRMZM2G043417*	*GRMZM2G075683*	*GRMZM2G146283*	0.63	0.31	0.31	0.32
*GRMZM2G009598*	*GRMZM2G101635*	*GRMZM2G331368*	0.63	0.16	0.29	0.34
*GRMZM2G009598*	*GRMZM2G156818*	*GRMZM2G040247*	0.63	0.12	0.32	0.35
*GRMZM2G111022*	*GRMZM2G046932*	*GRMZM2G008226*	0.63	0.24	0.28	0.34
*GRMZM2G010797*	*GRMZM2G064145*	*GRMZM2G401050*	0.63	0.18	0.32	0.34
*GRMZM2G110402*	*GRMZM2G040207*	*GRMZM2G119703*	0.63	0.25	0.32	0.34
*GRMZM2G009598*	*GRMZM2G101635*	*GRMZM2G123660*	0.63	0.17	0.28	0.32
*GRMZM2G009598*	*GRMZM2G138425*	*GRMZM2G091151*	0.63	0.30	0.32	0.33
*GRMZM2G153792*	*GRMZM2G179024*	*GRMZM2G072894*	−0.63	−0.20	−0.33	−0.35
*GRMZM2G009598*	*GRMZM2G101635*	*GRMZM2G097068*	0.63	0.19	0.29	0.33

**Fig. 2:**
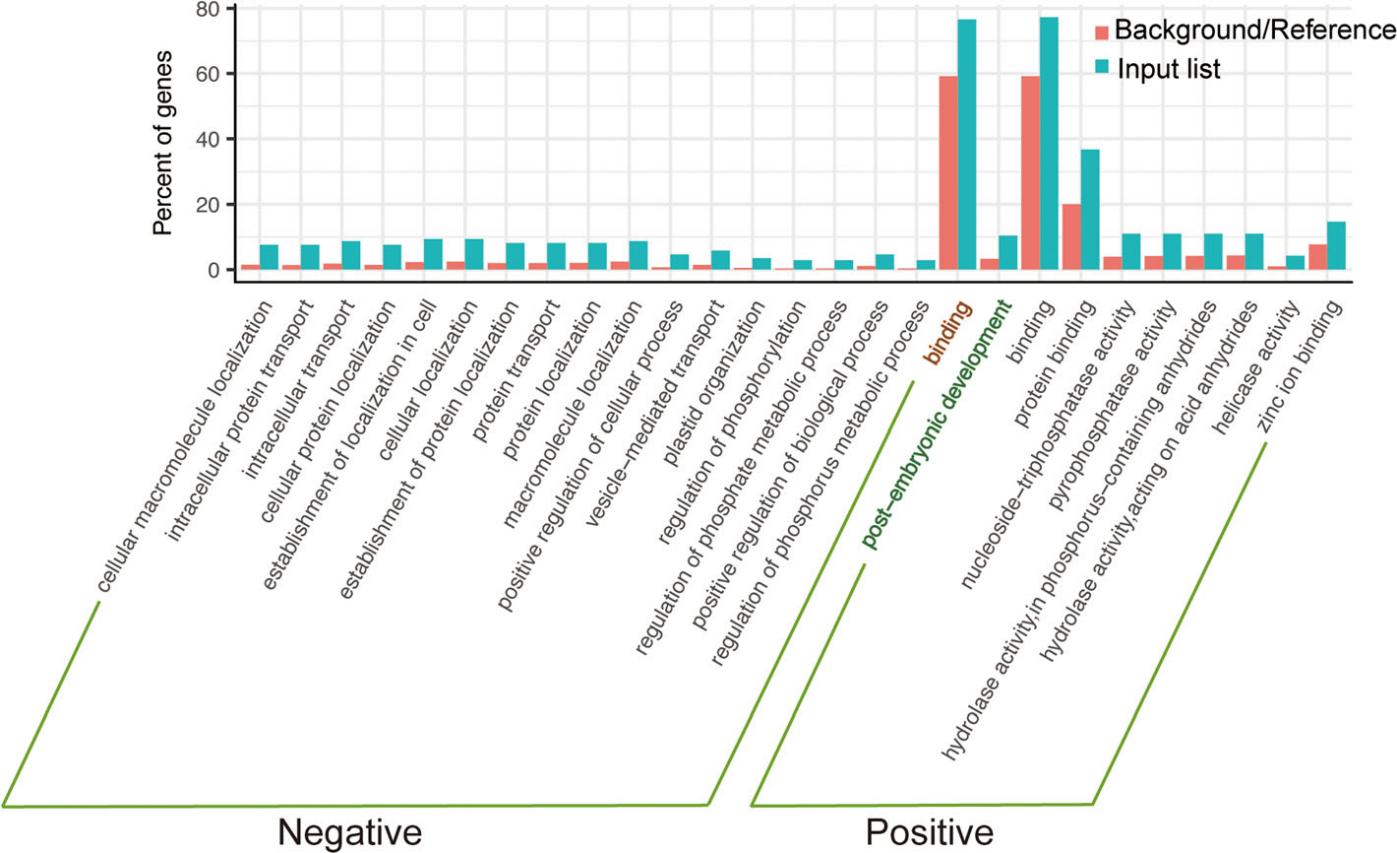
GO analysis of the top 200 LA-scouting leaders. Enrichment analysis of GO annotation for the top 200 LA-scouting leaders with negative and positive LA scores

LA-scouting leaders represent the LA-scouting genes with the highest LA-scouting ability. Using a stringent cutoff of at least 5.00 × 10^5^ linkages per LA-scouting gene, we identified 830 LA-scouting leaders based on positive LA scores and 215 LA-scouting leaders based on negative LA scores (Fig. 1). The Gene Ontology (GO) analysis of the top 200 LA-scouting leaders with negative LA scores indicated that they are significantly enriched in some biological processes, especially in positive regulation of cellular process and regulation of phosphorus metabolic process (Fig. 2 and Additional file 7: Figure S3a), and the same GO analysis were also remarkably enriched in molecular function of binding activities (Fig. 2 and Additional file 7: Figure S3b). In addition, the top 200 LA-scouting leaders with positive LA scores were involved in some molecular functions, such as nucleoside-triphosphatase, pyrophosphatase and hydrolaseactivity (Fig. 2 and Additional file 8: Figure S4a), and the same GO analysis were also remarkably enriched in some biological process, especially in post-embryonic development process (Fig. 2 and Additional file 8: Figure S4b). These results showed that phosphorus activities are involved in both positive and negative LA scores.

### Dynamic co-expression pattern of gene pairs in the carotene biosynthetic pathway

**Fig. 3:**
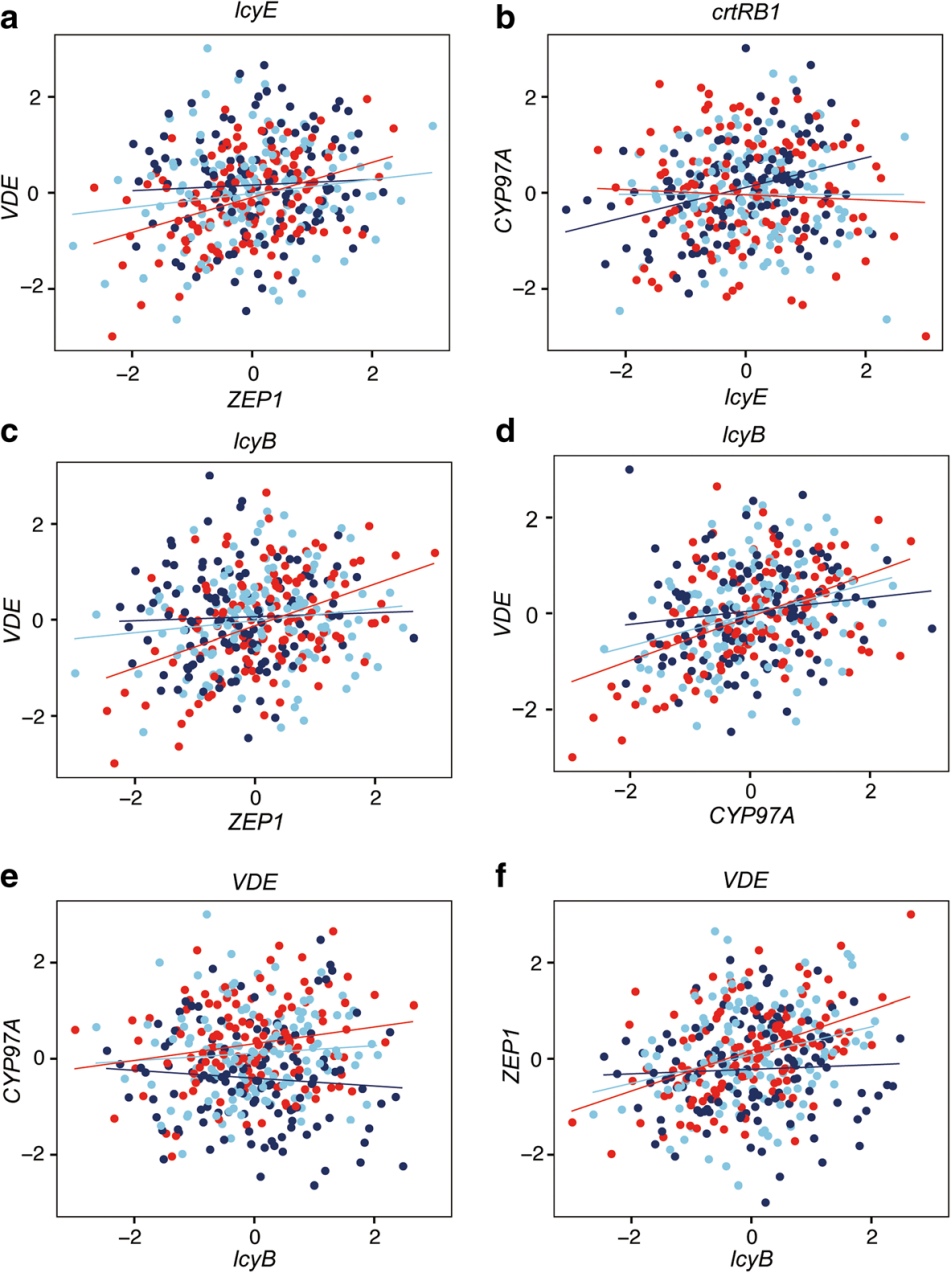
Dynamic co-expression analysis of gene pairs in the carotene biosynthetic pathway. **a**–**f** Co-expression patterns of LAPs are mediated by expression of the third genes. Each red dot indicates a maize line in which the expression of the LA-scouting gene (shown above each scatter plot**)** is high, a dark blue dot indicates a maize line in which LA-scouting gene expression is low, and a light blue dot indicates a maize line in which LA-scouting gene expression is moderate. The corresponding correlations are shown with matching colors. **a** Co-expression pattern of *ZEP1* and *VDE* is mediated by expression of *lcyE*. **b** Co-expression pattern of *lcyE* and *CYP97A* is mediated by expression of *crtRB1*. **c** Co-expression pattern of *ZEP1* and *VDE* is mediated by expression of *lcyB*. **d** Co-expression pattern of *CYP97A* and *VDE* is mediated by expression of *lcyB*. **e** Co-expression pattern of *lcyB* and *CYP97A* is mediated by expression of *VDE*. **f** Co-expression pattern of *lcyB* and *ZEP1* is mediated by expression of *VDE*

Dynamic co-expression analysis revealed that a gene pair for the β-carotene branch of the carotene biosynthesis pathway, *ZEP1* and *VDE*, is significantly linked to *lcyE*, which encodes lycopene ε-cyclase. When expression of *lcyE* was high, we found a strong positive correlation between *ZEP1* and *VDE* expression. In contrast, when expression of *lcyE* was low, the correlation between *ZEP1* and *VDE* expression dropped nearly to zero (Fig. 3a and Table 2). The regulatory role of *lcyE* on β-carotene branch biosynthesis is consistent with a previous study [25].

**Table 2:** Dynamic co-expression patterns of carotene biosynthesis genes

*X*	*Y*	*Z*	Corr_high^a^	Corr_low^b^	LA	*P* value
*ZEP1*	*VDE*	*lcyE*	0.34	0.05	0.21	9.00 × 10^−5^
*lcyE*	*CYP97A*	*crtRB1*	−0.06	0.33	−0.20	4.30 × 10^−4^
*ZEP1*	*VDE*	*lcyB*	0.38	0.04	0.23	2.00 × 10^−5^
*CYP97A*	*VDE*	*lcyB*	0.43	0.14	0.24	5.00 × 10^−5^
*lcyB*	*CYP97A*	*VDE*	0.17	−0.08	0.24	5.00 × 10^−5^
*lcyB*	*ZEP1*	*VDE*	0.43	0.05	0.23	4.00 × 10^−5^

^a^The correlation between *X* and *Y* under high *Z* expression

^b^The correlation between *X* and *Y* under low *Z* expression

Another LAP, *lcyE* and *CYP97A*, was significantly linked to *crtRB1*, which encodes β-carotene hydroxylase. When expression of *crtRB1* was high, the correlation between *lcyE* and *CYP97A* dropped to zero; however, with low *crtRB1* expression, the correlation between *lcyE* and *CYP97A* was significantly positive (Fig. 3b and Table 2). Similarly, the dynamic co-expression patterns for two additional LAPs (*ZEP1* and *VDE*, *CYP97A* and *VDE*) were significantly linked to *lcyB*, which encodes lycopene β-cyclase, the enzyme responsible for cyclizing lycopene to β-carotene (Fig. 3c-d and Table 2) [26]. Higher expression of *lcyB* was associated with a stronger positive correlation between *ZEP1* and *VDE*, whereas low expression of *lcyB* corresponded with the disappearance of the correlation between *ZEP1* and *VDE*. Similar results were found for the LA analysis of *CYP97A* and *VDE*, which suggests that *lcyB* has a substantial role in mediating violaxanthin biosynthesis.

There are five genes in the violaxanthin biosynthesis pathway: *lcyB*, *CYP97A*, *ZEP1*, *crtRB1* and *VDE*. With dynamic co-expression analysis, we also found similar co-expression patterns between *lcyB* and *CYP97A* and between *lcyB* and *ZEP1* given changes in the expression of *VDE* (Fig. 3e-f and Table 2). Conditioning on the high *VDE* expression, we saw clear positive co-expression patterns between *lcyB* and *CYP97A*, and the co-expression pattern between *lcyB* and *ZEP1* was also positively correlated. Our results have provided new insight into the regulation of *lcyB*, *CYP97A*, *ZEP1* and *VDE*, which may change the direction of carotene biosynthesis by controlling the influx and efflux of the substrate.

### Dynamic trait-trait correlation patterns in the oil biosynthetic pathway

**Fig. 4:**
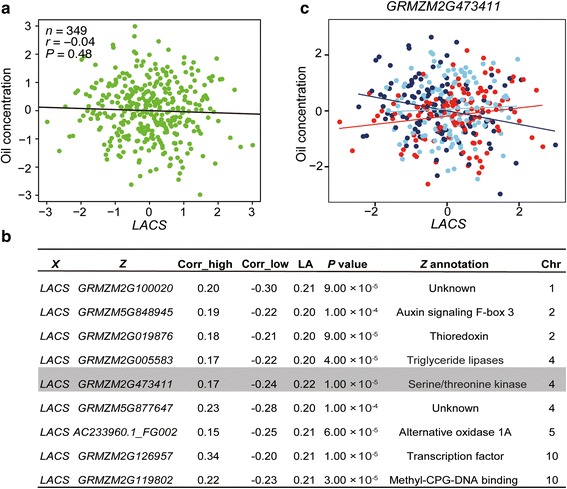
Co-expression pattern of *LACS* and oil concentration is mediated by nine genes**. a** The correlation between *LACS* and oil concentration. **b** Nine genes that mediate the co-expression pattern of *LACS* and oil concentration. **c** The co-expression pattern of *LACS* and oil concentration at different expression levels of *GRMZM2G473411*. Each red dot indicates a maize line in which *GRMZM2G473411* expression is high, a dark blue dot indicates a maize line in which *GRMZM2G473411* expression is low, and a light blue dot indicates a maize line in which *GRMZM2G473411* expression is moderate. The corresponding correlations are shown with matching colors

A previous GWA study identified 26 loci significantly associated with oil concentration, and candidate gene association analyses of some of those genes found indels (insertions and deletions) in their 3’untranslated regions (UTRs) or non-coding regions, suggesting that regulation at the level of transcription leads to natural variation in oil phenotypes [8]. However, the expression of these candidate genes was not significantly correlated with the oil phenotypes [8]. We thus applied the LA methodology to these oil-associated genes, by using the expression of these 26 individual genes and oil concentration as the pair (*X*, *Y*) and any additional gene as *Z*. We computed LA scores as described above. We found that 22 oil-related genes were co-regulated with a *P* value = 1.00 × 10^−4^conditioning on 482 LA-scouting genes and 17 oil-related genes were contra-regulated conditioning on 127 LA-scouting genes, including 9 oil-related genes that were both co- and contra-regulated. In total, 23 oil-related genes were correlated with oil concentration mediated by 580 unique LA-scouting genes. We annotated these 580 unique LA-scouting genes and found that about 29 genes were implicated in lipid metabolism in *Arabidopsis thaliana* or other species (Additional file 9: Figure S5). The proteins encoded by the remaining 551 genes were classified as signaling molecules, stress responsers, transcription factors, and enzymes involved in biological pathways including oxidation-reduction reactions, and protein metabolism. The functions of approximately one-third of the identified genes are currently unknown (Additional file 9: Figure S5). In the example of *LACS* (*GRMZM2G079236*), previous re-sequencing results identified two completed linked indels (indel_146 and indel_472) in the 3’UTR that were significantly associated with oil concentration [8]. However, the expression levels between *LACS* and normalized oil concentration were uncorrelated (*r* = −0.04, *P* = 0.48, *n* = 349; Fig. 4a). By carrying out a dynamic correlation analysis of gene expression and oil concentration, we found that the trait-trait correlation patterns between *LACS* and oil concentration change as the expression levels of nine LA-scouting genes change (Fig. 4b). Among these LA-scouting genes, *GRMZM2G473411*, which had the highest LA score, encodes serine/threonine kinase, which is involved in a number of fundamental cellular processes such as ATP binding, oxidoreductase activity, protein kinase activity [27]. A strong negative correlation between the expression of *LACS* and oil concentration was found when expression of *GRMZM2G473411* was low (Fig. 4c). However, when *GRMZM2G473411* expression was high, there was a positive correlation between *LACS* expression and oil concentration (Fig. 4c). These results implied that *GRMZM2G473411* has a role in mediating oil biosynthesis.

We subsequently determined the regulatory network among 23 oil-related genes and LA-scouting genes (Additional file 10: Figure S6). The results indicated that *GRMZM2G122767*, which encodes a protein involved in ATP binding, is linked to 89 LA-scouting genes (Additional file 11: Table S5). For example, *GRMZM2G122767* and oil concentration were co-regulated conditioning on *GRMZM2G102878* with LA = 0.25 (Additional file 12: Figure S7a). When the expression level of *GRMZM2G102878* was high, a strong positive correlation was identified between the expression of *GRMZM2G122767* and oil concentration, and this correlation disappeared when the expression of *GRMZM2G102878* was low. *GRMZM2G102878*, which encodes fatty acyl-acyl carrier protein (ACP), functions as a substrate in the fatty acid synthesis pathway [28, 29]. A similar interpretation can be made based on the LA for *GRMZM2G122767* and oil concentration, with *GRMZM2G046804* being the mediator gene (Additional file 12: Figure S7b). When *GRMZM2G046804* expression was high, a strong positive correlation was identified between the expression of *GRMZM2G122767* and the oil concentration, whereas the correlation dropped nearly to zero when the expression of *GRMZM2G046804* was low. *GRMZM2G046804* encodes glyceraldehyde-3-phosphate dehydrogenase (GAPDH), which plays an important role in the glycolysis pathway [30, 31]. These results indicated that upstream genes in the metabolic pathways have a substantial role in mediating the oil biosynthesis pathway.

## Discussion

It is well known that all biological processes are interconnected, and many proteins have important roles in multiple cellular processes. Two proteins involved in a common process under certain conditions may function in independent processes under other conditions, which implies that both the strength and pattern of association between the expression profiles of two genes may vary as the intrinsic cellular-state changes [32]. These results in subtle co-expression patterns of two genes that are hard to recognize by standard similarity analyses based on correlation. In the literature, different measures such as structural equation models, Bayesian networks and other probabilistic graphical models are widely used for study conditional correlation and causal relationship [33–35]. However, many complex regulatory in the biological system can’t be captured by direct guilt-by association using above methods because of multi-way interaction [36]. For example, two gene expression levels are overall non-correlated but they exhibited high correlation when a third gene is high expressed and a much lower correlation when expression of the third gene is low. In this case, the third gene may serve as an indicator of certain cellular state or regulator that controls the presence and absence of co-regulation between two gene pairs [36]. To identify the conditional association in the gene triplet, Li (2002) proposed a liquid association to explore the dynamic-pattern as opposed to the static-pattern of gene expression in cell, and previous studies and our results has proved that LA method is a useful tool for investigating the dynamic nature of co-expression on a genome-wide scale [18, 20–22].

We conducted a genome-wide search and identified the significant critical cellular players that may affect the co-expression pattern of any two genes. We found 24,907 LA-scouting genes after filtering in this study. The LA result, with the size of the resulting dataset is 1.10 × 10^12^, represent a huge amount of data that can be sorted and organized in a variety of ways to meet different researchers’ needs. In this study, we focused on a small portion of the high-scoring LAPs. In general, a higher LA score is associated with a more obvious LA pattern when the profile plots are visually examined. It is in this sense that the leading LA-scouting genes are better surrogates of the relevant intrinsic cellular-state variables. But how we use these surrogates to infer the cellular state depends on the available biological knowledge [21]. Ultimately, our results can contribute to the understanding about the biological roles of maize genes, of which the vast majority are still not well characterized. Recently, a new method named LANDD (Liquid Association for Network Dynamics Detection) probably improved the interpretability of the results, which find subnetworks with concentrated LA relationships. This method used the collective behaviour of genes in a subnetworks as indicators of cellular states rather than one gene [37]. LAPs with high LA scores are likely to be involved in biological pathways (Additional file 6: Table S4), which implies that metabolism-related genes are more susceptible to being regulated in this manner. Of course, with rapid accumulation of transcript omic studies, combining multiple studies to indentifying LA triplets is likely to produce more accurate and stable results [36].

The LA system is useful for predicting the functions of little known genes. For example, *GRMZM5G858880* is an LA-scouting gene with a high positive LA score. Characterization of *GRMZM5G858880* has been quite limited, and its functional annotation is vaguely worded as “encode WW domain-containing protein” by MaizeGDB (MazieGenome Database). Among the list of LAPs for *GRMZM5G858880* (Additional file 13: Table S6), we found several genes involved in ribosome protein synthesis, translation initiation, and protein phosphorylation: *GRMZM2G092663* (ribosomal protein S5 family protein, appearing three times), *GRMZM2G099352* (ribosomal protein S3 family protein), *GRMZM2G168149* (ribosomal protein S5 family protein), *GRMZM2G092663* (ribosomal protein S5 family protein), *GRMZM2G129015* (ribosomal protein S26e family protein, appearing twice), *GRMZM2G164352* (protein phosphatase 2A subunit A2, appearing four times), *GRMZM2G122135* (protein phosphatase 2A subunit A2, appearing twice), *GRMZM2G064133* (eukaryotic translation initiation factor 3G1). This is consistent with the *GRMZM5G858880* homolog in *Arabidopsis*, which regulates translation through two broad mechanisms: ribosomal stalling and reducing re-initiation efficiency [38].

We were also able to demonstrate the applicability of LA using the maize data on the carotene biosynthetic pathway. We show how the transition from α-carotene to β-carotene is mediated by a delicate switch between the co-expression and contra-expression of *CYP97A* and *lcyE*. This switching mechanism depends on the expression of *crtRB1*. A previous study has shown that high expression of *lcyE*, which is a key gene in the β-carotene branch of the pathway, is conducive to the accumulation of β-carotene [39]. Researches also validated that *crtRB1* transcripts accumulated to a greater extent in lines with low β-carotene amounts relative to lines with high β-carotene [4]. In our LA results, expression of *CYP97A* and *lcyE* was positively correlated when the expression of *crtRB1* was low, which suggested that both the substrate and energy were flowing into the β-carotene branch. Knowledge of the entire pathway and an understanding of the key genes involved at each step in the pathway allow the manipulation of the pathway to create maize grain with higher levels of provitamin A (Pro-VA) content. For instance, the dynamic co-expression patterns of *PSY1*, *lcyE*, and *crtRB1* were tested in this study. The LA analysis indicated that the levels of *lcyE* and *crtRB1* expression were slightly positively correlated when the expression of *PSY1* was high, *PSY1* expression and *crtRB1* expression were slightly negatively correlated when *lcyE* expression was low, and *PSY1* expression and *lcyE* expression were also slightly negatively correlated when the expression of *crtRB1* was low (Additional file 14: Table S7). These results agree with findings from previous studies that upregulation of *PSY1* and co-downexpression of *lcyE* and *crtRB1* correspond to the high level of natural variation for Pro-VA components [4, 6]. Thus the dynamic co-expression patterns of key genes in biosynthetic pathways can be used to guide the selection of gene combinations for more efficient biofortification by marker-assisted selection and genetic modification.

For quantitative traits, a substantial proportion of phenotypic variation can be explained by differences in gene expression. Re-sequencing analysis found indels (some very long) in the UTRs or promoter regions are significantly associated with oil concentration in 4 of 26 oil-related genes in maize, potentially accounting for gene expression differences seen in the RNA-seq results [8]. Unexpectedly, expression of these four candidate genes does not correlate with the corresponding traits based on a statistical analysis. Here we developed a new application for LA by taking the oil concentration as variable *Y* to find the correlation between gene expression level and phenotype. From the LA results in the oil biosynthetic pathway, 23 of 26 oil-associated genes were correlated with oil concentration conditioning on 580 individual LA-scouting genes. Among these 580 LA-scouting genes, only 5% were directly involved in the oil biosynthesis pathway, whereas the others encoded regulatory factors or enzymes involved in biological pathways that potentially regulate the oil biosynthetic pathway according to the LA results. Although additional functional verifications of these LA-scouting genes are needed, the LA method provides a new perspective for understanding the genetic architecture and genetic regulation of oil biosynthesis and accumulation.

## Conclusions

The LA method provided an effective way to dissect dynamic trait-trait correlation patterns and identified significant critical cellular players in carotene and oil biosynthesis in maize kernels. We carried out a genome-wide LA analysis and found that some biological pathways were notably enriched for these LAPs and LA-scouting genes. The LA analysis in the carotene biosynthetic pathway revealed relationships among several important genes that can change the flow from α-carotene to β-carotene by a delicate switch between co-expression and contra-expression. The application of the LA method to analyze the oil biosynthesis pathway indicated the presence of a genetic regulatory mechanism at the level of transcription. Future work is needed to assess the biological roles of LA-scouting genes and to extend the LA system for analyses of more complex correlations.

## Methods

### Data

We carried out our LA analysis using an RNA-seq dataset of 28,769 annotated genes sequenced from kernels collected 15 days after pollination from 368 maize lines [11]. The expression values for each gene were normalized using a normal quantile transformation with the qqnorm function in R [11]. Missing values were imputed with average expression values. The oil concentration phenotypes from the 368 genotypes, which have been described in detail previously [8], were used for the LA analysis within one pathway. The phenotypic values for each line were also normalized using a normal quantile transformation with the qqnorm function in R [11].

### Theory of LA analysis

The LA theory is presented in terms of continuous random variables [21]. Li proposed the concept of LA to describe how the co-expression pattern of two genes, *X* and *Y*, changes according to the level of a third gene, *Z*. Suppose all variables are standardized to have mean 0 and variance 1. So the correlation between variables *X* and *Y* is equal to *E*(*XY*). Conditioning on a third variable *Z*, the conditional expectation *E*(*XY|Z = z*) is denoted by *g*(*z*) so that the overall correlation between *X* and *Y*, *E*(*XY*) = *Eg*(*Z*). *g*(*z*) is regarded as the co-expression measure between gene pair *X* and *Y* when *Z* is expressed at level *z*. The derivative *g*’(*z*) quantifies how *g*(*z*) varies as *z* increases. If *Z* is continuous random variable, change of the conditional expectation can be described by its derivative. The definition of LA:

$$ \mathrm{LA}\ \left(X,Y|Z\right)=E\left({g}^{\prime }(Z)\right) $$

where

$$ \mathrm{g}(Z)=E\left( XY|Z=z\right) $$

when the *Z* is standard normal,

$$ \mathrm{LA}\ \left(X,Y|Z\right)=E(XYZ) $$

So a normal score transformation on each gene profile is performed before analysis.

### Genome-wide LA analysis

We used the statistical method LA to measure dynamic co-expression patterns [20, 21]. The LA method describes the intuitive change in the co-expression of a pair of genes, *X* and *Y*. If the state change turns out to be associated with the differential expression of a third gene, *Z*, then the profile of *Z* can be used to screen the scatter plot of (*X*, *Y*) for LA activity. If an increase in *Z* is associated with an increase in the correlation of (*X*, *Y*), then gene *Z* is a positive LA-scouting gene for (*X*, *Y*), and a positive score is assigned to quantify the strength of LA. The pair (*X*, *Y*) is called a positive LAP of *Z*. Similarly, a negative LA-scouting gene is defined as an increase in *Z* that is associated with a decrease in the correlation of (*X*, *Y*), and the LA score of the LAP is negative. Consequently, when the low and high expression levels of a negative LA-scouting gene are compared, the scouted LAP is likely to change from being co-expressed to being contra-expressed. For a positive LA-scouting gene, the change goes in the opposite direction, from contra-expression to co-expression [21].

For the genome-wide LA study, we standardize each gene-expression profile with a normal score transformation firstly. Then the average product of the three transformed profiles was computed as follow,

$$ \mathrm{LA}\ \mathrm{score}=\left({X}_1{Y}_1{Z}_1+{X}_2{Y}_2{Z}_2+\cdots +{X}_m{Y}_m{Z}_m\right)/\mathrm{m} $$

where *m* is the total number of maize inbred lines.

### Genome-wide LA significance assessment

To determine if a LA linkage score is statistically significant or not, we generated a reference distribution of LA scores under the assumption of no linkage using a simulation scheme. At each permutation, we randomly sample two expression values (cells) in the matrix as *X* and *Y* and re-compute the LA scores for all other genes as *Z* and then record the most positive and most negative values. We repeated this procedure 1.00 × 10^6^ times and obtained the reference distributions for the positive LA scores and the negative LA scores. Then we also generated a genome-wide LA score distribution. At each genome-wide LA analysis, we randomly sample genes (rows) in the matrix as *X* and *Y* and re-compute the LA scores for all other genes as *Z* and also recode the most positive and most negative values. We repeated this procedure 1.00 × 10^6^ times and obtained the genome-wide LA score distributions for the positive LA scores and the negative LA scores. We compared the genome-wide LA score distribution with the reference distribution by a quantile-to-quantile plot. Next we estimate the FDR value by assuming the proportion of null cases is 100%. Suppose there are altogether N gene pairs under consideration and the permutation *P* value (the quantile of the reference distribution) cutoff is *p*. We calculate the false discovery rate: FDR = *Np*/*D*, where *D* is the number of gene pairs with permutation *P* values ≤ *p* [40, 41].

### Definition of LA-scouting genes in genome-wide significant LA triplets

Within the significant LA triplet, we used MLA to determine the LA-scouting gene [42]. When the conditional means and variances also depend on *X*_*3*_, MLA can measure liquid association using *ρ*(*X*_1_, *X*_2_| *X*_3_) as the co-expression measure of *X*_*1*_ and *X*_*2*_ given *X*_*3*_ instead of *E*(*X*_1_, *X*_2_| *X*_3_) : *h*(*X*_3_) = *ρ*(*X*_1_, *X*_2_| *X*_3_). So, MLA represents the expected value of the change of the conditional correlation with *X*_*3*_. The definition of MLA applied Stein’s lemma [43]:

$$ \mathrm{MLA}\ \left({X}_1,{X}_2|{X}_3\right)=E\left\{{h}^{\prime}\right.\left({X}_3\left.\Big)\right\}\right.=E\left\{h\right.\left({X}_3\left.\Big){X}_3\right\}\right. $$

A direct estimate for MLA is:

$$ \widehat{\mathrm{M}\mathrm{LA}}=\frac{\sum_i^M\widehat{\rho_i}\overline{X_{3i}}}{\mathrm{M}} $$

where M is the number of bins over *X*_*3*_, $\widehat{\rho}$_*i*_ is the sample Pearson’s correlation coefficient of *X*_*1*_ and *X*_*2*_ using only those observations with *X*_*3*_ in bin *i*, and $\overline{X}$_*3i*_ is the mean of *X*_*3*_ in bin *i*. The total number of bines M is set to 3 in all MLA estimations throughout the analysis.

### Gene function annotation and GO enrichment analysis

To more fully explore the functions of candidate genes, the annotation resources of maizeGDB (http://maizecyc.maizegdb.org) and the InterPro (http://www.ebi.ac.uk/interpro/) database were integrated into the analyses [44, 45]. GO enrichment analyses were performed using the agriGO tool (http://systemsbiology.cau.edu.cn/agriGOv2/) with SEA (Singular Enrichment Analysis) option [46, 47]. Hypergeometric distributions were applied to test the significance against the maize genome background, and the *P* values were adjusted for multiple testing by controlling the FDR. The updated GO items of the maize genome were downloaded from Ensembl BioMarton April 4, 2013 [48].

### LA analysis within one pathway

Thirteen genes involved in the carotenoid biosynthetic pathway were selected from MaizeGDB were used for computing the triplet combinations using LA theory. And we defined the LA-scouting gene in significant LA triplet based on biological significance. 26 oil concentration–associated genes identified in a previous GWA study were considered as *X*, and the oil concentration phenotype normalized by the qqnorm function in R was considered as *Y* [8]. A genome-wide gene-trait LA was computed across all the 24,907 genes. As the genome-wide LA significance score is strict, we used a local permutation method. Briefly, we randomly permuted *Z* genes as many as 1 million times and computed their LA scores. The *P* values represent how often the permuting LA scores exceeded the estimated LA score [21].

## Additional files

Additional file 1: Table S1. The permutation test for positive and negative LA thresholds. (XLSX 12 kb)

Additional file 2: Figure S1. Quantile-quantile plot of genome-wide negative (a) and positive (b) LA scores versus randomly generated LA scores. (DOCX 121 kb)

Additional file 3: Table S2. The number of LAPs with expression mediated by the LA-scouting leaders (*P* values <1.00 × 10^−5^). (XLSX 844 kb)

Additional file 4: Figure S2. *P* value versus FDR. (DOCX 1655 kb)

Additional file 5: Table S3. Quantile-based permutation thresholds for *P* values, LA scores and FDRs. (XLSX 10 kb)

Additional file 6: Table S4. Annotations of the top 50 triplets with the highest LA scores. (XLSX 16 kb)

Additional file 7: Figure S3. GO analysis of the top 200 LA-scouting leaders with negative LA scores. (DOCX 1896 kb)

Additional file 8: Figure S4. GO analysis of the top 200 LA-scouting leaders with positive LA scores. (DOCX 1609 kb)

Additional file 9: Figure S5. Functional category annotations for 580 LA-scouting genes linked to oil-associated genes. (DOCX 13 kb)

Additional file 10: Figure S6. The regulatory network that includes 23 oil concentration–associated genes and their linked LA-scouting genes. The network comprises 23 nodes and 609 edges. The thickness of the lines indicates the value of the LA and each pink and green dot represents a *Z* and *X* gene, respectively. (DOCX 74 kb)

Additional file 11: Table S5. The LA results for *X* = *GRMZM2G122767* with significant LA scores in oil biosynthesis. (XLSX 17 kb)

Additional file 12: Figure S7. Dynamic co-expression patterns of *GRMZM2G122767* and oil concentration are mediated by G*RMZM2G102878* and *GRMZM2G046804*. **a** Each red dot indicates a maize line in which *GRMZM2G102878* expression is high, a dark blue dot indicates a maize line in which *GRMZM2G102878* expression is low, and a light blue dot indicates a maize line in which *GRMZM2G102878* expression is moderate. **b** Each red dot indicates a maize line in which *GRMZM2G046804* expression is high, a dark blue dot indicates a maize line in which *GRMZM2G046804* expression is low, and a light blue dot indicates a maize line in which *GRMZM2G046804* expression is moderate. (DOCX 340 kb)

Additional file 13: Table S6. The LA results for *Z* = *GRMZM5G858880* with significant LA scores in the oil biosynthetic pathway. (XLSX 10 kb)

Additional file 14: Table S7. The LA results for *PSY1*, *lcyE*, and *crtRB1* in the carotene biosynthetic pathway. (XLSX 9 kb)

## Abbreviations

ACP: Acyl-acyl carrier protein
DGAT: Diacylglycerol Acyltransferase
e-QTL: Expression quantitative trait locus
FDR: False discovery rate
GAPDH: Glyceraldehyde-3-phosphate dehydrogenase
GO: Gene ontology
GWA: Genome-wide association
Indels: Insertions and deletions
LA: Liquid association
LAP: Liquid association pair
MAF: Minor allele frequency
TAG: Triacylglycerol
UTR: Untranslated region
