# Supplementary material for: Genome-wide trait-trait dynamics correlation study dissects the gene regulation pattern in maize kernels
Source: BMC Plant Biol. 2017 Oct 16;17:163. doi: 10.1186/s12870-017-1119-y (PMC5644097; doi:10.1186/s12870-017-1119-y)
Supplement: Supplementary file 2 — Quantile-quantile plot of genome-wide negative (a) and positive (b) LA scores versus randomly generated LA scores. (DOCX 121 kb) [file 12870_2017_1119_MOESM2_ESM.docx]

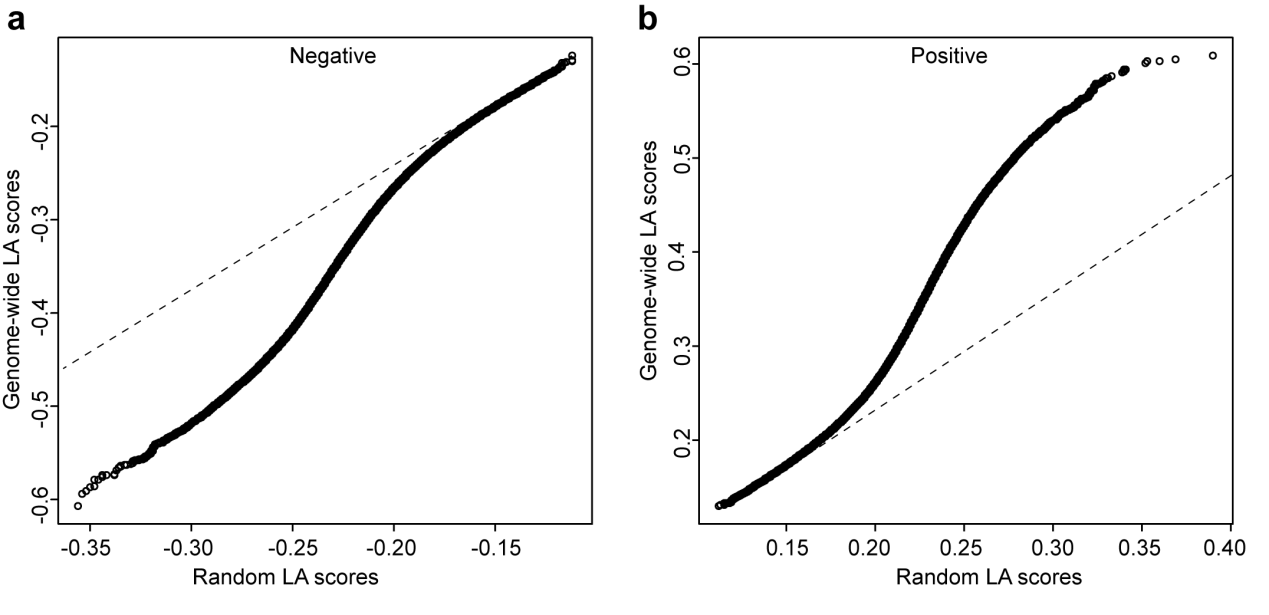


**Fig.S1:**Quantile-quantile plot of genome-wide negative (a) and positive (b) LA scores versus randomly generated LA scores.
