# Supplementary figures and images for: Genome-wide trait-trait dynamics correlation study dissects the gene regulation pattern in maize kernels
Source: BMC Plant Biol. 2017 Oct 16;17:163. doi: 10.1186/s12870-017-1119-y (PMC5644097; doi:10.1186/s12870-017-1119-y)

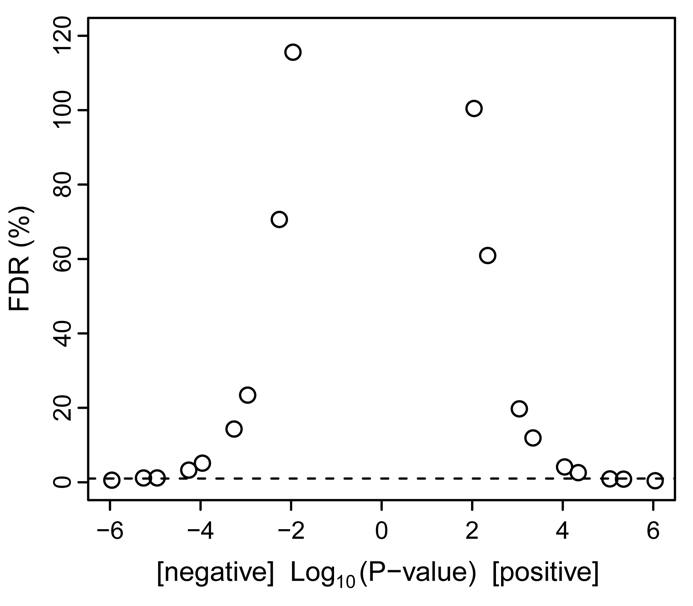


**Fig.S2**: *P* value versus FDR.

Supplement: Supplementary file 4 — P value versus FDR. (DOCX 1655 kb) [file 12870_2017_1119_MOESM4_ESM.docx]
