# Supplementary material for: Genome-wide trait-trait dynamics correlation study dissects the gene regulation pattern in maize kernels
Source: BMC Plant Biol. 2017 Oct 16;17:163. doi: 10.1186/s12870-017-1119-y (PMC5644097; doi:10.1186/s12870-017-1119-y)
Supplement: Supplementary file 7 — GO analysis of the top 200 LA-scouting leaders with negative LA scores. (DOCX 1896 kb) [file 12870_2017_1119_MOESM7_ESM.docx]

**
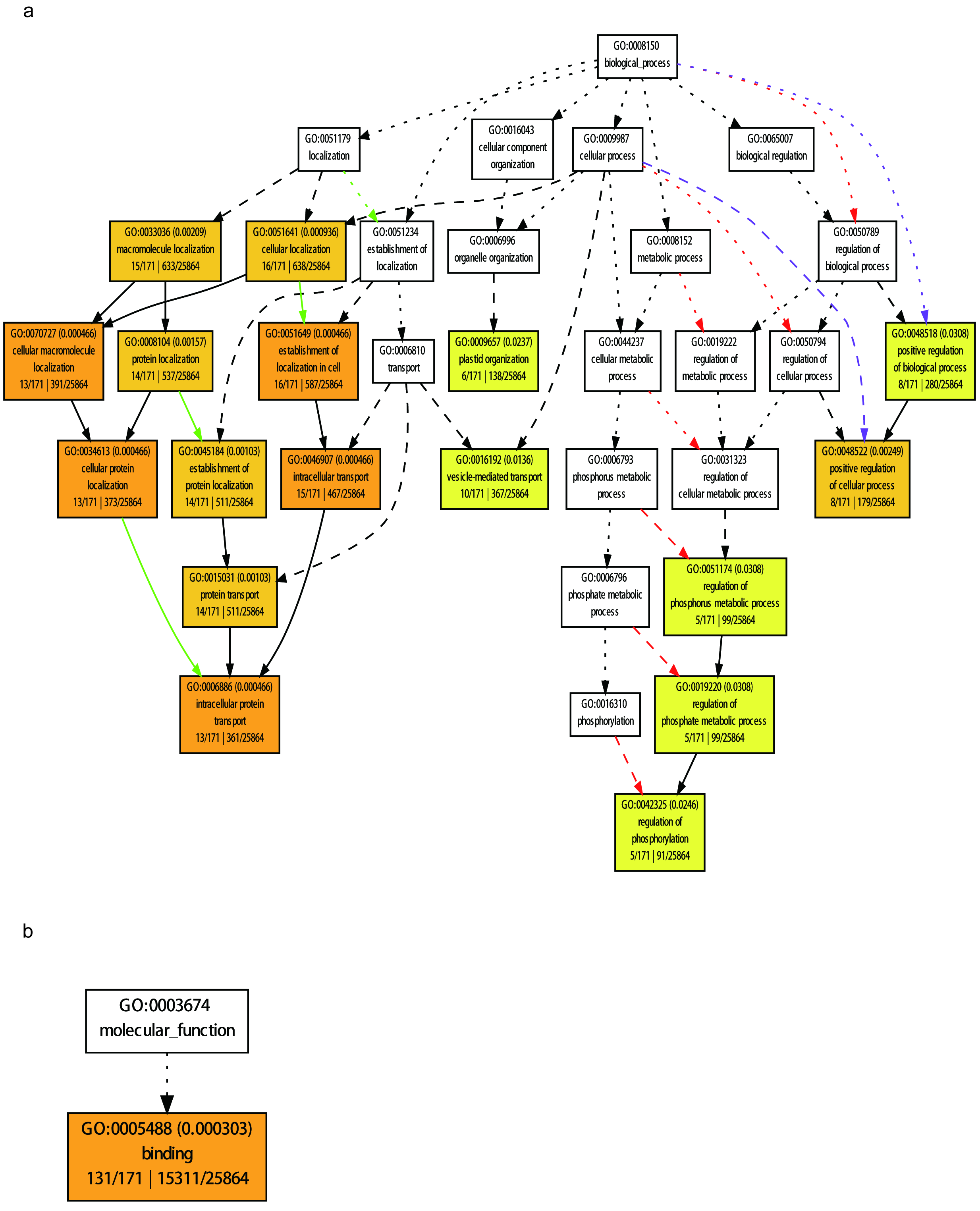
**

**Fig.S3** The GO analysis of the top 200 LA-scouting leaders with negative LA scores. **a** showing enrichment in some biological processes, **b** showing enrichment in molecular function.
