# Supplementary material for: Genome-wide trait-trait dynamics correlation study dissects the gene regulation pattern in maize kernels
Source: BMC Plant Biol. 2017 Oct 16;17:163. doi: 10.1186/s12870-017-1119-y (PMC5644097; doi:10.1186/s12870-017-1119-y)
Supplement: Supplementary file 8 — GO analysis of the top 200 LA-scouting leaders with positive LA scores. (DOCX 1609 kb) [file 12870_2017_1119_MOESM8_ESM.docx]

**
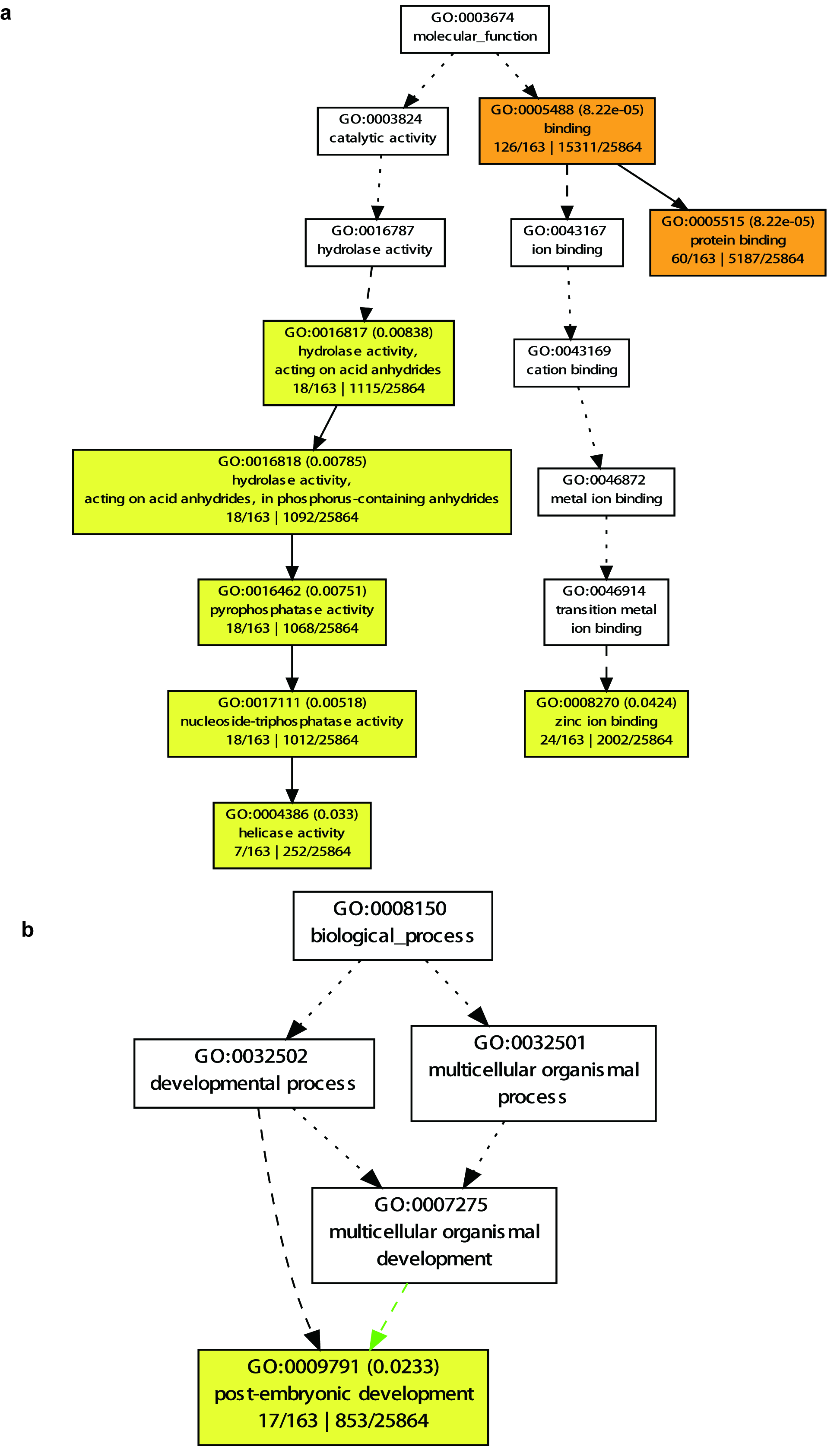
**

**Fig.S4** The GO analysis of the top 200 LA-scouting leaders with positive LA scores

**a** showing enrichment in molecular functions, **b** showing enrichment in biological processes.
