# Supplementary material for: Genome-wide trait-trait dynamics correlation study dissects the gene regulation pattern in maize kernels
Source: BMC Plant Biol. 2017 Oct 16;17:163. doi: 10.1186/s12870-017-1119-y (PMC5644097; doi:10.1186/s12870-017-1119-y)
Supplement: Supplementary file 9 — Functional category annotations for 580 LA-scouting genes linked to oil-associated genes. (DOCX 13 kb) [file 12870_2017_1119_MOESM9_ESM.docx]

**Fig.S5** Functional category annotations for 580 LA-scouting genes linked to oil-associated genes.
