# Supplementary material for: Genome-wide trait-trait dynamics correlation study dissects the gene regulation pattern in maize kernels
Source: BMC Plant Biol. 2017 Oct 16;17:163. doi: 10.1186/s12870-017-1119-y (PMC5644097; doi:10.1186/s12870-017-1119-y)
Supplement: Supplementary file 10 — The regulatory network that includes 23 oil concentration–associated genes and their linked LA-scouting genes. The network comprises 23 nodes and 609 edges. The thickness of the lines indicates the value of the LA and each pink and green dot represents a Z and X gene, respectively. (DOCX 74 kb) [file 12870_2017_1119_MOESM10_ESM.docx]

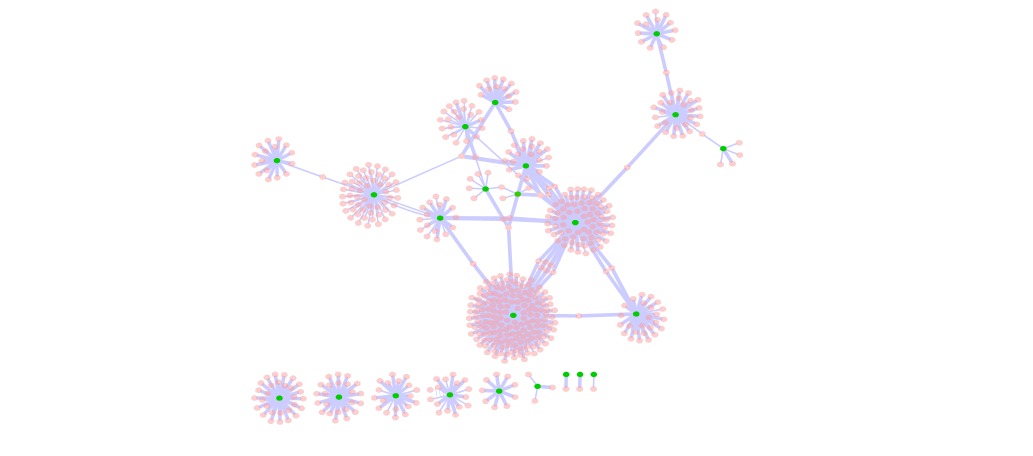


**Fig. S6** The regulatory network between 23 oil associated genes and their linked LA-scouting genes. The network comprises 23 nodes and 609 edges. The thickness of the line indicates the value of the LA and each pink and green dot represents a *Z* and *X* gene, respectively.
