# Supplementary material for: Genome-wide trait-trait dynamics correlation study dissects the gene regulation pattern in maize kernels
Source: BMC Plant Biol. 2017 Oct 16;17:163. doi: 10.1186/s12870-017-1119-y (PMC5644097; doi:10.1186/s12870-017-1119-y)
Supplement: Supplementary file 12 — Dynamic co-expression patterns of GRMZM2G122767 and oil concentration are mediated by GRMZM2G102878 and GRMZM2G046804. a Each red dot indicates a maize line in which GRMZM2G102878 expression is high, a dark blue dot indicates a maize line in which GRMZM2G102878 expression is low, and a light blue dot indicates a maize line in which GRMZM2G102878 expression is moderate. b Each red dot indicates a maize line in which GRMZM2G046804 expression is high, a dark blue dot indicates a maize line in which GRMZM2G046804 expression is low, and a light blue dot indicates a maize line in which GRMZM2G046804 expression is moderate. (DOCX 340 kb) [file 12870_2017_1119_MOESM12_ESM.docx]

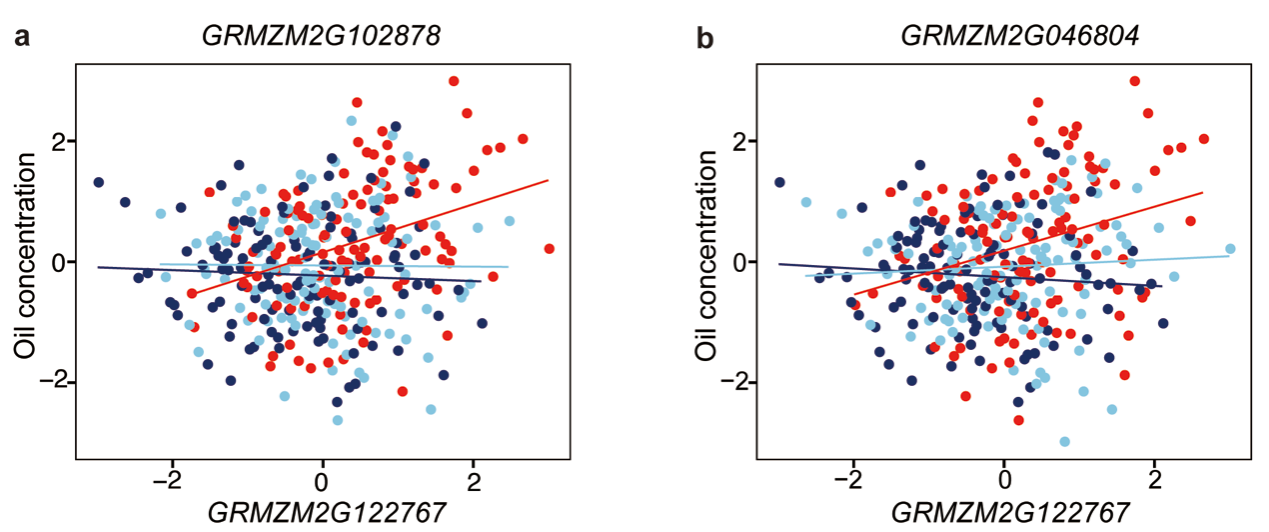


**Fig.S7** Dynamic co-expression patterns of *GRMZM2G122767* and oil concentration are mediated by G*RMZM2G102878* and *GRMZM2G046804*. **a** Each red dot indicates a maize line in which *GRMZM2G102878* expression is high, a dark blue dot indicates a maize line in which *GRMZM2G102878* expression is low, and a light blue dot indicates a maize line in which *GRMZM2G102878* expression is moderate. **b** Each red dot indicates a maize line in which *GRMZM2G046804* expression is high, a dark blue dot indicates a maize line in which *GRMZM2G046804* expression is low, and a light blue dot indicates a maize line in which *GRMZM2G046804* expression is moderate.
